# Label-Free Quantitative Phosphoproteomics of the Fission Yeast *Schizosaccharomyces pombe* Using Strong Anion Exchange- and Porous Graphitic Carbon-Based Fractionation Strategies

**DOI:** 10.3390/ijms22041747

**Published:** 2021-02-09

**Authors:** Barbara Sivakova, Jan Jurcik, Veronika Lukacova, Tomas Selicky, Ingrid Cipakova, Peter Barath, Lubos Cipak

**Affiliations:** 1 Institute of Chemistry, Slovak Academy of Sciences, Dubravska cesta 9, 845 38 Bratislava, Slovakia; chembsiv@savba.sk (B.S.); peter.barath@savba.sk (P.B.); 2 Biomedical Research Center, Cancer Research Institute, Slovak Academy of Sciences, Dubravska cesta 9, 845 05 Bratislava, Slovakia; jan.jurcik@savba.sk (J.J.); tomas.selicky@savba.sk (T.S.); ingrid.cipakova@savba.sk (I.C.); 3 Medirex Group Academy, n.o., Jana Bottu 2, 917 01 Trnava, Slovakia; Veronika.Lukacova@medirexgroup.sk

**Keywords:** LFQ phosphoproteomics, SAX, PGC, *Schizosaccharomyces pombe*

## Abstract

The phosphorylation of proteins modulates various functions of proteins and plays an important role in the regulation of cell signaling. In recent years, label-free quantitative (LFQ) phosphoproteomics has become a powerful tool to analyze the phosphorylation of proteins within complex samples. Despite the great progress, the studies of protein phosphorylation are still limited in throughput, robustness, and reproducibility, hampering analyses that involve multiple perturbations, such as those needed to follow the dynamics of phosphoproteomes. To address these challenges, we introduce here the LFQ phosphoproteomics workflow that is based on Fe-IMAC phosphopeptide enrichment followed by strong anion exchange (SAX) and porous graphitic carbon (PGC) fractionation strategies. We applied this workflow to analyze the whole-cell phosphoproteome of the fission yeast *Schizosaccharomyces pombe*. Using this strategy, we identified 8353 phosphosites from which 1274 were newly identified. This provides a significant addition to the *S. pombe* phosphoproteome. The results of our study highlight that combining of PGC and SAX fractionation strategies substantially increases the robustness and specificity of LFQ phosphoproteomics. Overall, the presented LFQ phosphoproteomics workflow opens the door for studies that would get better insight into the complexity of the protein kinase functions of the fission yeast *S. pombe*.

## 1. Introduction

Phosphorylation is a rapid, dynamic, and reversible post-translational modification that regulates the diversity of protein functions [1,2,3]. Despite the phosphorylation of proteins having an important regulatory function, the proteins also undergo nonspecific phosphorylation. As a result, certain levels of protein phosphorylation occur without any functional relevance. Additionally, for a given amino acid, the degree of phosphorylation, which reflects how many copies of a protein are phosphorylated at a particular amino acid at a given time point, might represent a threshold that distinguishes the relevant phosphorylation from the non-functional phosphorylation events. This makes the identification and the analysis of biologically relevant protein phosphorylation challenging [4,5,6].

The most powerful strategies to identify protein phosphorylation are mass spectrometry (MS)-based approaches. Due to their high throughput and sensitivity, the MS-based approaches have become golden standards in the field of phosphoproteomics [6,7,8,9]. Recently, label-free quantitative (LFQ) phosphoproteomics has become the most frequently used approach to analyze protein phosphorylation [10,11,12]. As opposed to MS-based phosphoproteomics strategies that require labeling of samples, such as stable isotope labelling by amino acids in cell culture (SILAC), isobaric tag labelling for relative and absolute quantitation (iTRAQ), or isobaric tandem mass tags (TMT) labelling, the LFQ phosphoproteomics has no general limits with regard to the number of analyzed samples or replicates [13,14,15].

Despite the robustness and continuous improvements of LFQ phosphoproteomics, the relatively low stoichiometry of some phosphoproteins limits the comprehensive analysis of protein phosphorylation [8]. Except for the prevention of phosphorylation and improved digestion efficiency in the proximity of the phosphorylated amino acid residues, the enrichment of samples with phosphopeptides is considered as the most critical step of phosphoproteomics [16,17,18]. Therefore, various phosphopeptide enrichment strategies have been developed. The most popular became metal-based methods, represented by immobilized metal affinity chromatography (IMAC) and metal oxide affinity chromatography (MOAC). While IMAC commonly uses metal cations (Fe^3+^, Ga^3+^, Zr^4+^, or Ti^4+^) as the affinity reagents for the negatively charged phosphate groups, the MOAC utilizes the affinity of oxygen in the phosphorylated groups for matrices that contain metal oxide. Titanium dioxide (TiO_2_) is the most commonly used MOAC reagent, followed by zirconia (ZrO_2_) and magnetite (Fe_3_O_4_). Both IMAC and MOAC enrich for phosphoserine, phosphothreonine, and phosphotyrosine peptides [19,20,21].

Besides the aforementioned preferences of IMAC and MOAC for the particular subsets of phosphopeptides, the success of phosphopeptide identification further relies on the recovery rate of the phosphopeptides, which may be influenced by the complexity of the samples. Therefore, the phosphopeptide-enriched samples are usually further processed by subsequent fractionations, such as strong cation exchange chromatography (SCX), strong anion exchange chromatography (SAX), porous graphitic carbon chromatography (PGC), or high pH reverse phase chromatography. For example, lowering the complexity of the samples by a basic SCX separation allowed the identification of more than 10,000 phosphopeptides [22]. Another study showed that SAX fractionation of Fe-IMAC-enriched samples allows identification of more than 14,000 phosphopeptides [23]. Similarly, PGC chromatography, which separates phosphopeptides based on their different hydrophobicity, led to the identification of several new phosphopeptides that were missed in the SCX and SAX fractioned samples [24,25].

**Figure 1:**
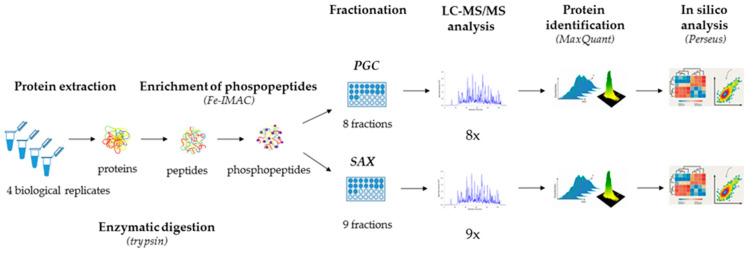
Workflow of label-free quantitative (LFQ) phosphoproteomics of the fission yeast *Schizosaccharomyces pombe*. The protocol involves the following steps: protein extraction, phosphopeptide enrichment (Fe-IMAC), PGC and SAX fractionations, LC-MS/MS analysis, protein identification, and in silico analysis.

Despite the great effort spent to optimize the LFQ phosphoproteomics analysis, there is still a need for its further advancement that would increase the robustness and identification rate. In this study, we present an optimized protocol for LFQ phosphoproteomics analysis of the whole-cell extract of the fission yeast *S. pombe*. Our LFQ phosphoproteomics workflow is based on Fe-IMAC phosphopeptide enrichment, followed by PGC and SAX fractionations of the phosphopeptides (Figure 1). Overall, the presented protocol requires only a small sample size (≤1.0 g of yeast cell pellet), and from the yeast cells collection to the bioinformatics analysis it takes less than 3 days. Importantly, the protocol can be parallelized with other phosphopeptide enrichment and fractionation strategies, which might further improve the identification of phosphopeptides.

Using this approach, we were able to detect 7079 annotated phosphosites and have identified an additional and so far non-annotated 1274 phosphosites, which represent a substantial addition to the phosphoproteome of the fission yeast *S. pombe*.

## 2. Results and Discussion

As the phosphorylation of proteins is a highly dynamic process, the preservation of protein phosphorylation through the protection of phosphomodifications and the abrogation of artificial phosphorylations are necessary. To preserve the in vivo phosphorylation status of our samples, the collected yeast cells were immediately snap-frozen in liquid nitrogen and grinded mechanically, as described previously [26]. Rapid snap-freezing is known to protect the proteins from hydrolysis and degradation, and prevents the changes in the phosphorylation status of the proteins [27,28]. Snap-freezing is also considered the quickest way to preserve the post-translational modifications in the samples and the best method of storing samples as long as the samples are placed into liquid nitrogen immediately after collection [29].

The first prerequisite to the effective phosphoproteomics analysis is efficient protein extraction. Thus, we compared here the conditions of protein extraction by re-suspending the yeast cell powder in a urea-based buffer and in a sodium deoxycholate (SDC) buffer. We found that the protein extraction in the SDC buffer was more efficient (21.37 µg/µL) compared to the urea-based buffer (12.78 µg/µL). It is known that urea is a strong chaotropic agent that denatures proteins by direct interaction with positively charged histidines. The subsequent formation of hydrogen bonds with polar amino acid residues lead to breaking down of the hydrophobic structures of the protein [30]. Alternatively, SDC is known to be an effective agent for the extraction of proteins. However, several studies pointed out that SDC suppresses the MS ion signals of the peptides. Therefore, the SDC must be removed from the samples before MS analysis [31,32,33]. Despite the better solubility and the higher concentration of proteins in samples extracted into the SDC buffer, we have detected about 14% more unique peptides and 6% more protein groups in the samples solubilized with the urea-based buffer compared to samples solubilized in the SDC buffer. Considering the need to remove the SDC before MS analysis and the identified higher number of peptides and proteins in samples extracted into the urea-based buffer, we further processed into our LFQ phosphoproteomics workflow only the samples extracted into the urea-based buffer. Following this, we stabilized the samples by reduction and alkylation and mixed them with trypsin in a 1:30 ratio (enzyme:protein). Trypsin is the most commonly used protease in proteomics and phosphoproteomics with a specificity to cleave the carboxyterminal of the lysine and arginine residues, resulting in a positive charge at the peptide C-terminus [34]. The use of a relatively high concentration of trypsin was shown to compensate for the reduced trypsin digestion efficiency in the proximity of the phosphorylated amino acid residues [35,36]. Alternatively, various proteases, such as Lys-C, Glu-C, Arg-C, Asp-N, Lys-N, chymotrypsin, or subtilisin, might be used for sequential digestion to improve the phosphoproteomics sampling depth [37,38,39]. As have been shown previously, using alternatives to tryptic digestion would enable the detection of phosphopeptides that stayed inaccessible by the trypsin-only digestion [40,41].

As many phosphopeptides have a substoichiometric abundance [8], the samples have to be enriched for phosphopeptides [16,17,18]. It has been demonstrated previously that phosphopeptide enrichment using Fe-IMAC offers a selective, comprehensive and reproducible phosphopeptide enrichment strategy [23,42]. Despite several studies pointing out that different phosphopeptide enrichment methods enrich samples with different populations of phosphopeptides [42,43], we stuck here only to the Fe-IMAC phosphopeptide enrichment to keep our LFQ phosphoproteomics workflow easily manageable.

Additionally, it is known that the complexity of the samples affects the recovery rates of the phosphopeptides [22,23,24,25]. To reduce the complexity of the samples and improve the efficiency of identification of phosphopeptides, high-performance liquid chromatography (HPLC) separation techniques have been shown as the highly efficient strategies [22,23,24,25,44,45]. For example, Lombardi et al. shown that basic SCX separation allows the identification of more than 10,000 phosphopeptides [22]. Similarly, Ruprecht et al. showed that lowering the complexity of samples by SAX fractionation allows identification of more than 14,000 phosphopeptides [23]. Additionally, the PGC chromatography helped to identify several new phosphopeptides that have been missed after SCX and SAX fractionations [24,25]. Altogether, these findings demonstrate the complementarity of various fractionation strategies and clearly stress out that the combination of various fractionation strategies greatly improves the efficiency of identification of phosphopeptides.

**Figure 2:**
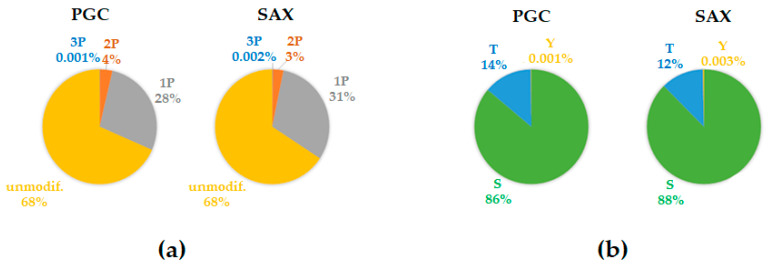
Phosphopeptide enrichment using the Fe-IMAC strategy. (**a**) The graph shows the average composition of the mono- (1P), double- (2P), and multi- (3P) phosphorylated peptides or unmodified peptides in separate PGC and SAX fractionation experiments. (**b**) The panel illustrates the percentage of individual phosphorylated amino acid residues (S-serine, T-threonine, and Y-tyrosine) in the modified peptides identified in the PGC and SAX fractionated samples.

As such, in our work we fractionated the Fe-IMAC-enriched samples using two fractionation strategies, PGC and SAX. To obtain feasible volumes of the samples for desalting, we pooled the SAX fractionated samples to nine fractions as opposed to eight PGC fractions from a smaller PGC column. The slightly different number of fractions collected from the first-dimension chromatographies was due to the different geometries and overall volumes of the columns used. The collected fractions were subsequently analyzed by high-performance liquid chromatography–tandem mass spectrometry and the raw data were analyzed using the quantitative proteomics software MaxQuant and Perseus (Figure 1). We found that Fe-IMAC enriched our sample for mono-phosphorylated peptides (28% and 31% for the PGC and SAX fractionated samples, respectively), followed by the di-phosphorylated peptides (4% and 3% for the PGC and SAX fractionated samples, respectively) and tri-phosphorylated peptides (0.001% and 0.002% for the PGC and SAX fractionated samples, respectively) (Figure 2a). Importantly, we observed no differences between the PGC and SAX fractionated samples in relation to the particular subsets of phosphopeptides (Figure 2b).

Detailed analysis of the PGC fractionated samples led to the identification of 2760 protein groups, 13,248 peptides, and 5149 phosphosites. The analysis of the SAX fractionated samples resulted in the identification of 2905 protein groups, 16,338 peptides, and 5875 phosphosites (Table 1).

In summary, 22,240 peptides belonging to 3139 proteins and 8353 phosphorylation sites were identified (Table S1). Combining the outputs of both fractionation techniques, we were able to increase the coverage of the *S. pombe* proteome by 7% (234 proteins that were only specifically found in PGC fractionations vs. 3139 total proteins) and to increase the coverage of the phosphoproteome by 30% compared to the SAX fractionations (2478 phosphosites that were only specifically found in the PGC fractionations vs. 8353 total phosphosites).

**Figure 3:**
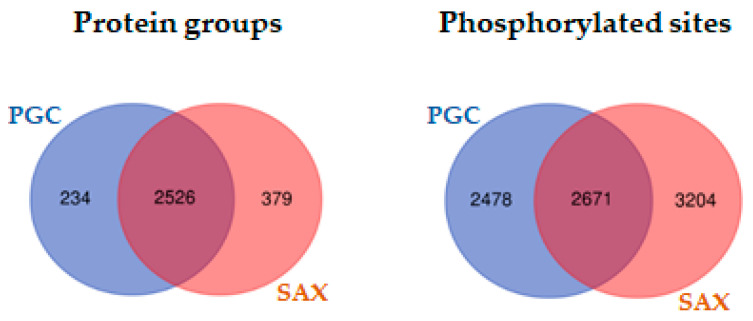
Venn diagrams. Comparison of the overlap between the identified phosphosites regarding the level of protein groups and phosphosites for samples fractionated by PGC and SAX.

Comparing the overlap of the sets of protein groups and phosphorylation sites identified after the PGC and SAX fractionations, we found that, among the 3139 protein groups identified by both fractionation techniques, 2526 protein groups were identical. In comparison, on the level of phosphosites only 2671 of the 8353 phosphosites were identified in samples fractionated by SAX or PGC. As such, in the case of protein groups, the value of the overlap was almost 80%. In contrast, the overlap on the level of phosphorylation sites identified in the SAX or PGC fractionated samples was significantly lower, only 32% (Figure 3).

Importantly, 1274 new phosphorylation sites, which had not been previously annotated, were identified (search against the *S. pombe* reference *PomBase* database, https://www.pombase.org (accessed on 7 December 2020)); 662 of these phosphosites were unique to PGC fractionation and 407 phosphosites were unique to SAX fractionation. This represents a substantial addition to the phosphoproteome of the fission yeast *S. pombe* (Table S1).

**Figure 4:**
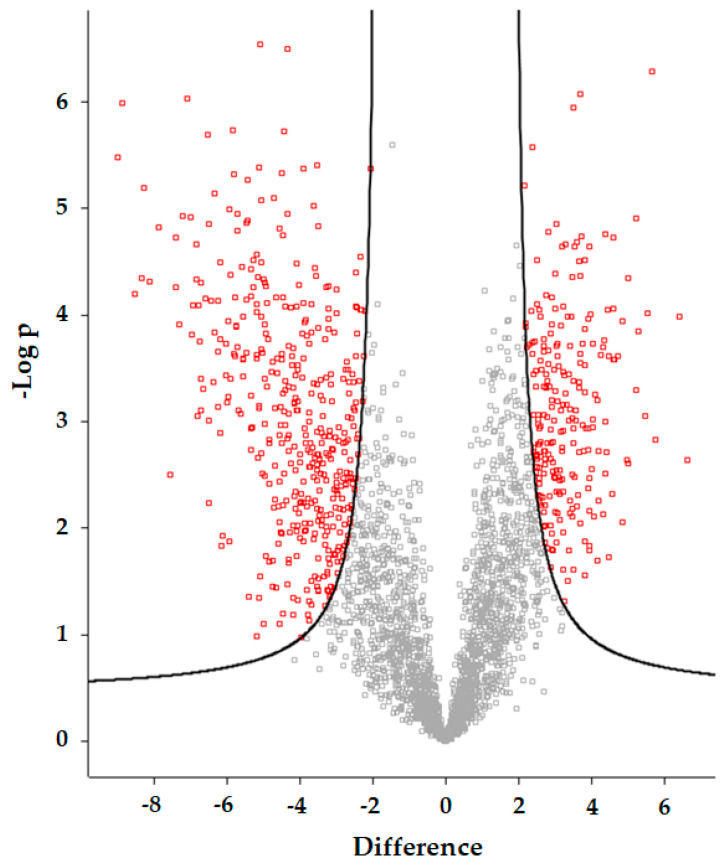
Volcano plot analysis of the differences in the identified protein groups between the PGC and SAX fractionated samples. Protein groups were filtrated using the following parameters: minimal valid values: *3*; mode: in at least one group. As a statistical test, a *t-*test with FDR = 0.01 and s0 = 2 was used. The red squares represent the protein groups whose intensities were >2-fold changed, compared to the intensity of the protein group identified by PGC and SAX.

To characterize the differences between the PGC and SAX fractionations in detail, we first analyzed the phosphoproteomics data at the level of protein groups using a Volcano plot analysis, with a *t-*test (FDR < 0.01, s0 = 2). After filtration of the protein groups for the valid values, we identified 2986 protein groups in total. Among them, 655 protein groups were significantly different for the PGC and SAX samples (red squares, Figure 4). All data, including the results of each specific statistical test (-log *p-*value), are in Table S2.

**Figure 5:**
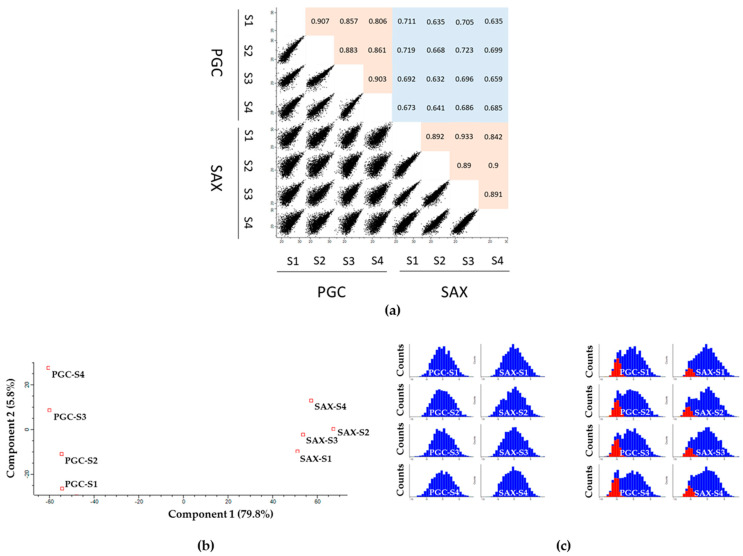
Overview of the correlation between the PGC and SAX fractionated samples and the distribution of the phosphopeptide intensities. To analyze the correlation between samples, (**a**) the MultiScatter plot and (**b**) the Principal Component Analysis (PCA) were used. In the case of the MultiScatter plot, the samples correlate more if the value of the Pearson correlation is close to 1 (red) and the comet has a narrow shape. For the PCA, the closer the samples are, the stronger their positive correlation is. (**c**) The distribution of the phosphopeptide intensities in each sample (marked in blue) was investigated using histograms. The missing values that were imputed from the Gaussian distribution are marked in red.

To further validate the correlation of phosphoproteomics data, we used a MultiScatter plot and Principal Component Analysis (PCA). In the case of the MultiScatter plot, correlation coefficients were calculated using a Pearson correlation for every Scatter plot. The range of Pearson correlation coefficients for the biological replicates prepared by the same fractionation technique were 0.806–0.907 for the PGC and 0.842–0.9 for the SAX fractionations; on the other hand, for replicates prepared by different techniques, this was in the range of 0.632–0.723. Additionally, the PCA enabled us to determine the degree of correlation between samples. The samples were measured in four biological replicates for each fractionation type and segregated based on component 1 and component 2, which accounted for 79.8% and 5.8% of the variability, respectively. Both the MultiScatter and PCA analyses showed that individual replicates prepared by the same fractionation method correlate more than samples prepared at the same time but processed by a different fractionation technique (Figure 5a,b). By the means of histograms, we also visualized the distribution of the protein intensities identified in each sample and compared the overall intensity distribution for all samples. We found that the protein intensities in each sample had a Gaussian distribution (blue columns, Figure 5c). Therefore, we used the corresponding statistical test for data with a normal distribution and imputed the missing values using Perseus. The extent of the imputations was examined by displaying the imputed values in the histogram. As expected, the imputed values fitted to the area of the low abundance protein groups (red columns, Figure 5c).

**Figure 6:**
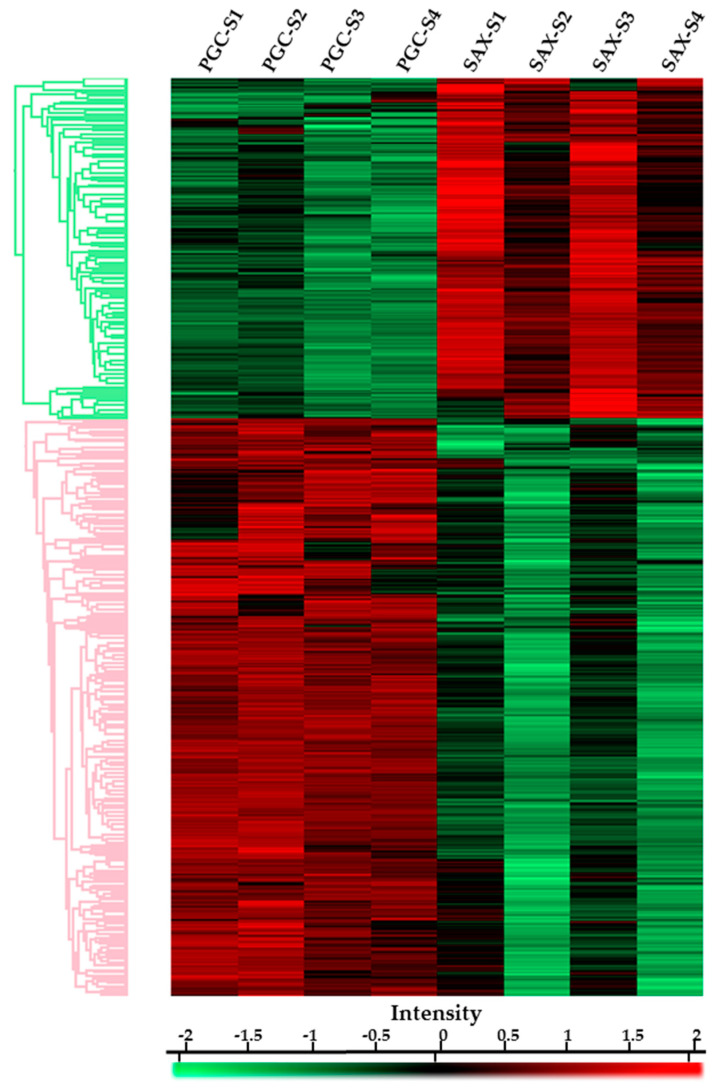
Heat map of the identified phosphosites. The heat map represents the significantly different phosphorylation sites identified after PGC or SAX fractionations. The phosphosites with increased intensities are marked in red, and phosphosites with lower intensities are marked in green.

To characterize the differences among the obtained phosphosites identified after the PGC and SAX fractionations, we analyzed the data by a statistical ANOVA test (permutation-based FDR ˂ 0.05). We found that, among the 6251 phosphorylation sites identified by PGC and SAX, 2875 phosphorylation sites differ significantly. Specific statistic details (-log ANOVA *p-*values) for the individual phosphosites are in Table S2. After Z-score normalization, using hierarchical clustering, we visualized the phosphosites in a Heat map (Figure 6), where the particular row belongs to a corresponding phosphorylation site, and a different color represents the magnitude of intensity. The scale from green to red indicates an increase in the intensity of the respective phosphorylation site. As presented, the identified phosphosites created two clusters (*green* and *pink*), according to their increased or decreased intensities in the PGC or SAX fractionated samples. In the case of the PGC fractionated samples, the intensities were increased in 1801 sites, and for samples prepared by SAX fractionation, the intensities were increased in 1065 phosphosites.

Using the Fisher exact test (B-H FDR < 0.02), we further analyzed the enrichment of the annotations (Gene Ontology terms, linear motifs for kinases) in the group of the significant ANOVA-tested phosphosites against all phosphosites. We found that the PGC fractionated samples are enriched with the *Raf1 kinase substrate motif* (enrichment factor, EF = 1.6, *p* = 0.3 × 10^−2^) and *Phosphorylase kinase substrate motif* (EF = 1.2, *p* = 0.15 × 10^−2^). The highest values of the enrichment factor were detected for samples prepared by SAX fractionation with the *BARD1 BRCT domain-binding motif* (EF = 1.8, *p* = 0.29 × 10^−3^) and *PAK2 kinase substrate motif* (EF = 1.6, *p* = 0.94 × 10^−3^). In the case of GO terms in the PGC fractionated samples, the most enriched biological processes were the *Regulation of GTPase activity* (EF = 1.6, *p* = 1.22 × 10^−5^) and the cell compartment *Cell division site* (EF = 1.5, *p* = 9.998 × 10^−5^). The SAX fractionated samples showed the highest value of the enrichment factor for *Cell septum* (EF = 2.0, *p* = 1.03 × 10^−5^) and *Cell cortex part* (EF = 1.6, *p* = 4.53 × 10^−7^) (Table S3).

The aforementioned enrichments of the PGC and SAX fractionations in GO terms are drawn from the genes known to be involved in regulation of cytokinesis, the process essential for cell growth, development, and differentiation [46,47,48]. In the fission yeast *S. pombe*, cytokinesis is tightly regulated by the septation initiation network (SIN). Insufficient SIN signaling results in improper assembly of the contractile ring and failure of the cytokinesis, generating multinucleated cells, whereas too much SIN signaling uncouples cytokinesis from the rest of the cell cycle [49]. As such, the cytokinetic events need to be precisely regulated to ensure that they occur in a proper and sequential manner [50]. Additionally, after the enrichment of the PGC and SAX fractionated samples for proteins involved in regulation of cytokinesis, we also identified multiple novel phosphorylation sites of proteins involved in cell division and cytokinesis (Table S1). In particular, we identified Rga2, a Rho2 GTPase-activating protein [51] to be phosphorylated on S127 and T989. Similarly, we identified novel S590 and S699 phosphosites for Rga6, and S3 phosphosite for Rga4, the well-known Rho2 GTPase-activating proteins [52,53]. Interestingly, the Cdc42 guanine exchange factor (GEF) Gef1, which activity is negatively regulated by conserved NDR kinase Orb6 [54], was identified to be additionally phosphorylated on T160 and S275. Moreover, we detected the core mitotic septin Spn1 to be phosphorylated on uncharacterized S17, and the protein kinase Sid2 or Ags1 to be phosphorylated on uncharacterized S19, S121, and S125, or S1136 and S1670, respectively. It has been shown previously that Spn1 in concert with SIN protein kinase Sid2 and the glucan synthases Bgs1 and Ags1 play an important role in the formation of a compact contractile actomyosin ring [55]. Altogether, the further studies of the biological role of the newly identified phosphomodifications and the identification of the protein kinases responsible for these modifications might have a great benefit in understanding of the regulation of various cellular processes.

In summary, we introduced here the optimized and easy-to-use strategy for LFQ phosphoproteomics analysis of the whole-cell extract of the fission yeast *S. pombe*. We showed that Fe-IMAC phosphopeptide enrichment followed by the PGC and SAX fractionations provides a reproductive and efficient strategy that enables detailed analysis of the *S. pombe* phosphoproteome in less than 3 days (Table 2).

Despite employing only a single type of phosphopeptide enrichment, we successfully detected 7079 annotated phosphosites and have identified an additional and so far non-annotated 1274 phosphosites of the fission yeast *S. pombe*. This represents a substantial addition to the phosphoproteome of the fission yeast *S. pombe*.

Importantly, the integration of alternatives to tryptic digestion and employment of additional phosphopeptide enrichment strategies into this workflow would offer a valuable option for even more comprehensive analysis of the *S. pombe* phosphoproteome. This would help us to better understand the regulatory functions of the particular protein kinases and might shed more light on the fundamental principles of the dynamics of protein phosphorylation.

## 3. Materials and Methods

### 3.1. Cell Culture and Protein Digest

The overnight culture of the fission yeast *S. pombe* strain (*h^-^*) was diluted in 1 L of YE + 5S media (5 g/L yeast extract, 30 g/L glucose, 0.15 g/L adenine, and 0.1 g/L each of uracil, L-histidine, L-lysine, and L-leucine) to OD_660_ = 0.15 and grown at 25 °C till OD_660_ = 0.8. The cells were collected by filtration using Magnetic Filter Funnels (Pall Corporation, MI, USA, cat.# 4242) and GN-6 47 mm 0.45 µm Metricel MCE Membrane Disc Filters (Pall Corporation, MI, USA, cat.# 66265), washed once with 100 mL ice-cold milli-Q H_2_O, and immediately frozen in a liquid nitrogen. The yeast cell powders were made by grinding yeast cells in the cryogenic grinder SPEX 6770 Freezer/Mill (SPEX SamplePrep, NJ, USA) [26]. One gram of the yeast cell powder was lysed in 1.5 mL ice-cold lysis buffer (8 M urea, 25 mM Tris-HCl, pH 8.5, 100 mM triethylammonium bicarbonate (TEAB), pH 8.5, and 150 mM NaCl) in the presence of phosphatase inhibitors (2.5 mM β-glycerol phosphate, 1 mM KF, 1 mM Na_3_VO_4_, and 1 mM Na_2_H_2_P_2_O_7_), or in 1.5 mL ice-cold 0.5% sodium deoxycholate buffer. Following this, the samples were vortexed and incubated at 200 *g* for 5 min at 60 °C. For reduction, DTT was added to the extracted samples to a final concentration of 5 mM and incubated for 30 min at 60 °C. Afterward, the samples were cooled to room temperature and alkylated with 40 mM chloroacetamide at 37 °C for 60 min. After five-fold dilution with 50 mM TEAB, pH 8.5, TPCK trypsin was added (1:30, *w*/*w*), and incubation for 16 h at 37 °C followed. The digestion was stopped by addition of 1% trifluoroacetic acid (TFA). Peptides were oxidized with 200 mM H_2_O_2_ and incubated for 30 min at 30 °C. The samples were then centrifuged at 14,100 *g* for 5 min at room temperature to pellet the precipitated lipids, and the peptides were desalted using C_18_ solid-phase extraction cartridges (Supelco, PA, USA, cat.# 57012). The desalted eluates were dried by vacuum centrifugation and the peptides were precipitated with ethyl acetate and dried repetitively.

### 3.2. Fe-IMAC Column Phosphopeptide Enrichment

For phosphopeptide enrichment, the sample was dissolved in 1 mL of Fe-IMAC solvent A (30% ACN, 0.07% (*v/v*) TFA) and loaded onto a Fe-IMAC column (9 × 50 mm ProPac IMAC-10, Thermo Scientific, MA, USA) connected to an FPLC chromatographic system (NGC Discover 100 Pro, Bio-Rad, CA, USA). Prior to use, the column was charged with iron ions. The flow rate of the system was 1 mL/min. After column connection to the FPLC system and initial equilibration for 9 min, the samples were applied (20 min run) and unbound peptides were washed out with Fe-IMAC solvent A for 32 min. Subsequently, phosphopeptides were eluted with a linear gradient from 0% to 45% Fe-IMAC solvent B (0.5% (*v*/*v*) NH_4_OH) for 48 min. After increase to 100% Fe-IMAC solvent B and a 5-min holding step, the column was re-equilibrated with Fe-IMAC solvent A for 32 min. Fractions were collected according to the UV signal (280 nm), and the concentration of peptides in individual fractions was measured by spectrophotometer DS-11 (DeNovix, DE, USA). The fractions were dried by vacuum centrifugation and dissolved in 1 mL of 1% TFA.

### 3.3. Porous Graphitic Carbon Separation

For separation of phosphopeptides to fractions, an analytical column (Hypercarb Porous Graphite Carbon LC column, 2.1 × 100 mm, Thermo Fisher Scientific, MA, USA) connected to an HPLC chromatographic system (Accela LC system, Thermo Fisher Scientific, MA, USA) was used. The flow rate of the system was 150 μL/min. After equilibration for 3 min with 100% Hypercarb solvent A (0.1% TFA), the samples were applied to the column and unbound peptides were washed out with 100% Hypercarb solvent A for 3 min, followed by elution with a linear gradient from 0% to 100% Hyperb solvent B (100% Acetonitrile) for 27 min. After an increase to 100% Hypercarb solvent B and a 10-min holding step, the column was washed out with Hypercarb solvent C (95% methanol) for 3 min. The fractions were collected in 1-min intervals. Based on the chromatogram, the sample was divided into 8 fractions, dried by vacuum centrifugation, and measured.

### 3.4. Hydrophilic Strong Anion Exchange Separation

For hydrophilic strong anion exchange (hSAX) separation, the Accela LC system (Thermo Fisher Scientific, MA, USA) equipped with a ProPac SAX-10 LC column (4 × 250 mm, Thermo Fisher Scientific, MA, USA, 054997) was used. The samples were dissolved in 100 μL of hSAX solvent A (5 mM Tris-HCl, pH 8.5). The flow rate of the system was 1 mL/min. After the sample application and subsequent equilibration with hSAX solvent A (3 min), the peptides were eluted with a linear gradient from 0% to 40% hSAX solvent B (5 mM Tris-HCl, pH 8.5, 1 M NaCl) for 17 min. After elution, hSAX solvent B was increased to 100% (10 min) and held constant for 10 min. A switch to 100% hSAX solvent A for 3 min was followed by column re-equilibration with 100% solvent A for 10 min. The fractions were collected in 1-min intervals. Based on the chromatogram, the sample was divided into 9 fractions, which were desalted using C_18_ solid-phase extraction cartridges (Sigma, Saint Louis, MO, USA, cat.# 504270), dried by vacuum centrifugation, and measured.

### 3.5. LC-MS/MS Analysis

LC-MS/MS analysis of phosphopeptides was performed on an LTQ Orbitrap Elite mass spectrometer (Thermo Fisher Scientific, MA, USA) equipped with Ultimate 3000 RSLC nano-HPLC system (Dionex, Germany) and nano-spray source (Thermo Fisher Scientific, MA, USA). Phosphopeptides were re-dissolved in 2% ACN/0.1% TFA and loaded onto a trap column (μ-Precolumn, 300 μm i.d. × 5 mm, C_18_ PepMap 100, 5 μm, 100 Å, Thermo Fisher Scientific, MA, USA) at a flow rate of 5 μL/min in 100% solvent A (0.05% TFA, 2% ACN in MS-grade water). The phosphopeptides were then transferred to an analytical column (Acclaim PepMap 100 C_18_ LC Column, 0.075 × 500 mm, Thermo Fisher Scientific, MA, USA) and separated using a concave gradient from 4% to 50% solvent B (0.08% FA, 80% ACN in MS-grade H_2_O) at a flow rate of 0.300 μL/min. The peptides were ionized using a 2.3-kV spray voltage and capillary temperature of 250 °C. The mass spectrometer was operated in a data-dependent acquisition mode using the Top15 strategy for the selection of precursor ions for the HCD fragmentation. An Orbitrap MS^1^ scan was taken (scan range, 300 to 1700 (m/z); resolution (R), 120K; max injection time, 10 ms), followed by ion trap MS^2^ scans on the top 15 peaks (minimum signal required, 4 × 10^4^ normalized units; higher-energy collisional dissociation energy, 25%; isolation width, 2 (m/z); default charge state, 2; activation time, 0.100 ms). For internal calibration, the signal at m/z 445.120030 was used as a lock mass. The mass spectrometry proteomics data have been deposited to the ProteomeXchange Consortium via the PRIDE [56] partner repository with the dataset identifier PXD023818.

### 3.6. Peptide and Protein Identification and Data Analysis

The resulting raw data files were processed using MaxQuant (v 1.5.3.30) with a built-in Andromeda search engine. The search was performed against the target-decoy version of the *Schizosaccharomyces pombe* UP2485 proteome FASTA (UniProt). The specific parameters for searching were carbamidomethyl (C) and oxidation (M) as a fixed modification, and N-terminal acetylation (protein N-term) and phosphorylation (S, T, Y) as variable modifications. Trypsin/P was specified as the proteolytic enzyme; the maximum number of missed cleavage sites permitted was two and the minimum peptide length required was six. The false discovery rate (FDR) of identification was estimated by searching a database with reversed sequences. The peptide and protein FDRs were all set to 0.01.

### 3.7. In Silico and Statistical Analysis

The statistical analysis of the MaxQuant output tables was performed using the Perseus software platform (v 1.5.5.3), which helps in the interpretation of protein quantification and post-translational modification data. Output tables from MaxQuant with protein groups and phosphosites were loaded, and the data were filtered for the reverse peptides, the contaminants, the peptides identified only by the site (protein groups), and for the localization probability of phosphosite greater than 75% (phosphosites). Filtering for valid values was also implemented; for further analysis, three valid values were needed in at least one group (PGC or SAX). After that, the visualization tools were used. The correlation between samples was investigated using MultiScatter plot, Principal Component Analysis, and Numeric Venn diagrams. A histogram was used for illustration of the distribution of protein intensities and range of imputations. Missing values were imputed using Perseus with downshift 1.8 and width 0.3. Identification of significantly differentially expressed proteins was performed by Volcano plot with a *t-*test (FDR < 0.01, S0 = 2). Differences on the level of phosphosites were examined using an ANOVA test (permutation-based FDR < 0.05). Gene ontology annotation lists, and linear motifs for the kinases are default settings in Perseus. After Z-score normalization, hierarchical clustering of the ANOVA significant phosphosites was used for visualization by Heat map. For annotation enrichment, Fisher’s exact test was used (B-H FDR < 0.02).

## Figures and Tables

**Table 1:** Summary of the protein groups, peptides, and phosphosites identified in particular samples after Fe-IMAC phosphopeptide enrichment followed by PGC and SAX fractionations.

Samples	Protein Groups		Peptides		Phosphosites	
	PGC	SAX	PGC	SAX	PGC	SAX
S1	2575	2820	10245	14509	3690	4629
S2	2581	2758	10813	13529	4213	4522
S3	2512	2753	9597	13909	3628	4802
S4	2463	2705	8855	12999	3628	4725
**Unique**	2760	2905	13,248	16,338	5149	5875
	**3139**		**22,240**		**8353**	

**Table 2:** Time-scale of individual steps of the LFQ phosphoproteomics workflow.

LFQ Phosphoproteomics Steps	Duration (h)
Denaturation, reduction, and alkylation	3
Tryptic digestion	16
Reversed-phase chromatography, ethyl acetate extraction	4
Fe-IMAC enrichment for phosphopeptides	5
PGC and SAX fractionation, reversed phase chromatography	3 (PGC)/9 (SAX)
LC-MS/MS analysis	14
Protein identification, in silico analysis	4
Total Time:	55

## Data Availability

The data presented in this study are openly available from the ProteomeXchange Consortium via the PRIDE partner repository with the dataset identifier PXD023818.

## Supplementary Materials

The following are available online at https://www.mdpi.com/1422-0067/22/4/1747/s1, Table S1: List of identified protein groups, peptides and phosphosites of the fission yeast *S. pombe*, Table S2: Results of Volcano plot analysis, Table S3: The values of intensities for identified phosphopeptides.
